# All‐cause mortality and Japan's early countermeasures

**DOI:** 10.1002/hsr2.1905

**Published:** 2024-02-15

**Authors:** Søren Roest Korsgaard

**Affiliations:** ^1^ Independent researcher Dar es Salaam Tanzania

A letter by Kaneda et al. in *Health Science Reports* argued that the Japanese government's early countermeasures to the World Health Organization‐declared coronavirus disease 2019 (COVID‐19) pandemic had failed.^1^ However, upon closer examination, this conclusion is questionable.

The conclusion was based in part on a paper by Wang et al., who found that Japan experienced significant excess mortality between January 2020 and December 2021.^2^ However, when using all‐cause mortality as the metric, the data actually suggest that the government's early countermeasures were among the most effective worldwide. Several studies have shown that Japan had a significant mortality deficit in 2020. A paper by Onozuk et al. quantified this deficit as 20,982 fewer deaths than expected (95% empirical confidence interval: −38,367 to −5472).^3^ Accordingly, life expectancy increased, while in many other countries it decreased.^4^

This is particularly striking given that the government did not impose a lockdown.^5^ In contrast, Italy, which imposed various mitigation measures, including lockdowns, experienced 100,526 excess deaths in 2020 (95% CI: 97,575–103,560) according to a study by Dorrucci et al.^6^ Several studies have found that the majority of countries experienced significant excess mortality in 2020.^7^, ^8^, ^9^ For example, a study of 29 high‐income countries by Islam et al. found that only three countries, New Zealand, Norway, and Denmark, experienced no excess mortality in 2020.^10^ The collective excess mortality was estimated to be 979,000 deaths (95% Cl: 954,000–1,001,000).

Despite its direct relevance, Kaneda et al. did not mention Japan's 2020 mortality deficit or its implications in their retrospective analysis of the government's early countermeasures. Clearly, the question must be asked: If the early countermeasures failed, as argued, how could there have been a mortality deficit in 2020? Any re‐examination of the early governmental countermeasures must put weekly, monthly, or at least yearly changes in all‐cause mortality into perspective and take into account the timing of any significant changes in government policy and the public's reaction to those changes.

Notwithstanding Japan's mortality deficit in 2020, mass vaccination with messenger RNA vaccines commenced in February 2021.^11^ At its peak, more than one million people were vaccinated daily. Three months later, contrary to expectations, deaths from all causes began to rise, eventually reaching an estimated 111,000 excess deaths (95% Cl: 103,000–116,000) by December 2021.^2^ According to a paper by Tanaka et al., analyzing Japanese age‐standardized mortality rates from 1995 to 2021, the excess mortality could be attributed to COVID‐19, cardiovascular disease, senility, and “other causes not classified as major causes.”^4^

The latter two causes largely accounted for the excess mortality in 2021. Regarding COVID, the Ministry of Health, Labour and Welfare registered 233,066 PCR‐positive severe acute respiratory syndrome coronavirus 2 cases and 3468 COVID‐attributed deaths in 2020. The global average of deaths and cases per million population in 2020 was several times higher.^12^ By December 31, 2021, these numbers had increased significantly to 1,727,984 and 18,385, respectively.^13^ According to a paper by Chowdhury et al., the increase in cases and deaths cannot be attributed to the Tokyo Summer Olympics, which took place from July 23 to August 8, 2021.^14^

Judging by all‐cause mortality, the Japanese government's response to COVID‐19 appears to have been among the most effective in 2020.

## AUTHOR CONTRIBUTIONS

**Søren Roest Korsgaard**: Conceptualization; investigation; writing—original draft; funding acquisition; methodology; validation; visualization; writing—review and editing; formal analysis; software; project administration; resources; supervision; data curation.

## CONFLICT OF INTEREST STATEMENT

The author declares no conflict of interest.

## TRANSPARENCY STATEMENT

The lead author Søren Roest Korsgaard affirms that this manuscript is an honest, accurate, and transparent account of the study being reported; that no important aspects of the study have been omitted; and that any discrepancies from the study as planned (and, if relevant, registered) have been explained.

## Data Availability

Data sharing is not applicable to this article as no new data were created or analyzed in this study.
